# MetaWorks: A flexible, scalable bioinformatic pipeline for high-throughput multi-marker biodiversity assessments

**DOI:** 10.1371/journal.pone.0274260

**Published:** 2022-09-29

**Authors:** Teresita M. Porter, Mehrdad Hajibabaei

**Affiliations:** Centre for Biodiversity Genomics @ Biodiversity Institute of Ontario & Department of Integrative Biology, University of Guelph, Guelph, ON, Canada; University of Hyogo, JAPAN

## Abstract

Multi-marker metabarcoding is increasingly being used to generate biodiversity information across different domains of life from microbes to fungi to animals such as for molecular ecology and biomonitoring applications in different sectors from academic research to regulatory agencies and industry. Current popular bioinformatic pipelines support microbial and fungal marker analysis, while ad hoc methods are often used to process animal metabarcode markers from the same study. MetaWorks provides a harmonized processing environment, pipeline, and taxonomic assignment approach for demultiplexed Illumina reads for all biota using a wide range of metabarcoding markers such as 16S, ITS, and COI. A Conda environment is provided to quickly gather most of the programs and dependencies for the pipeline. Several workflows are provided such as: taxonomically assigning exact sequence variants, provides an option to generate operational taxonomic units, and facilitates single-read processing. Pipelines are automated using Snakemake to minimize user intervention and facilitate scalability. All pipelines use the RDP classifier to provide taxonomic assignments with confidence measures. We extend the functionality of the RDP classifier for taxonomically assigning 16S (bacteria), ITS (fungi), and 28S (fungi), to also support COI (eukaryotes), rbcL (eukaryotes, land plants, diatoms), 12S (fish, vertebrates), 18S (eukaryotes, diatoms) and ITS (fungi, plants). MetaWorks properly handles ITS by trimming flanking conserved rRNA gene regions as well as protein coding genes by providing two options for removing obvious pseudogenes. MetaWorks can be downloaded from https://github.com/terrimporter/MetaWorks and quickstart instructions, pipeline details, and a tutorial for new users can be found at https://terrimporter.github.io/MetaWorksSite.

## Introduction

Marker gene sequencing, metabarcoding, or metasystematics are different terms for the same technique that involves extracting DNA from bulk samples such as soil, water, or mixtures of individuals collected from traps. One key strength of this technique is not having to isolate or identify individual specimens. A signature DNA region is then enriched, for example using PCR, to identify biological community composition using bioinformatics [1–3]. In microbial ecology to animal biodiversity studies, different signature DNA regions are chosen for their ability to identify target taxa. For example, in prokaryotes, the 16S small subunit (SSU) ribosomal RNA (rRNA) region is often used for genus level taxonomic assignments [4,5]. Other popular markers include cytochrome c oxidase (COI) for animals; ribulose bisphosphate large subunit (rbcL) for plants and diatoms; the internal transcribed spacer (ITS) for fungi and plants; 18S SSU for eukaryotes, arbuscular mycorrhizal fungi, and diatoms; and 12S mitochondrial SSU for fish [6–14].

Existing pipelines such as QIIME2 and DADA2 were initially developed to support the microbial ecology community [15,16]. In comprehensive, multi-trophic, multi-marker studies, there is a need for a pipeline that can handle rRNA genes, spacer regions, as well as protein-coding markers in a single harmonized environment [17,18]. For the ITS region, we needed a pipeline that could remove the conserved flanking rRNA genes as this has been shown to improve taxonomic assignment accuracy [19]. For protein-coding regions, we needed a pipeline that could remove putative pseudogenes [20–23]. We also wanted the ability to generate high quality exact sequence variants (ESVs) for popular metabarcoding markers (not just 16S or ITS) for the additional level of genetic and taxonomic resolution ESVs can provide [24–26]. For taxonomic assignment, we wanted to use a classifier that would provide a measure of confidence for assignments to reduce false-positive assignments [27–29].

As multi-marker studies are carried out on phylogenetically divergent taxa, such as in biodiversity or trophic studies, there is a need for more generic pipelines where different markers can be analyzed using similar dataflows with 3rd party programs instead of being limited to database-specific pipelines and tools [17,30]. We developed MetaWorks with the following objectives: 1) reproducibility with respect to the computational environment used as well as the pipeline itself, 2) scalability to leverage high performance computer clusters to speed up the analysis of large datasets, 3) naive Bayes classifier support for popular metabarcode markers; and 4) to support marker-specific processing steps such as ITS extraction and pseudogene-removal for protein-coding markers. MetaWorks was designed for data analysts who are comfortable using Linux command-line tools but would like a single harmonized environment and pipeline to process multi-marker metabarcode datasets.

## Implementation and workflow

### Implementation

MetaWorks is a multi-marker ‘meta’-barcode pipeline that does ‘the works’ by supporting the bioinformatic processing of popular markers including rRNA genes, spacers, and protein coding genes generating taxonomically assigned ESVs or operational taxonomic units (OTUs). To facilitate reproducibility, scalability, and shareability of workflows we use the Conda package manager to facilitate the download of most programs and dependencies and the Snakemake workflow manager to automate pipelines and utilize computational resources efficiently [31–33]. Snakemake supports re-entrancy and automatic deployment of multiple parallel jobs, both ideal for high performance computing environments where many cores are available to speed up the analysis of large datasets.

We provided instructions on how to install and use Conda in the online documentation. One additional program not available as a Conda package, ORFfinder, may need to be downloaded separately if pseudogene-filtering will be conducted and instructions are provided in the online documentation. MetaWorks can be downloaded from https://github.com/terrimporter/MetaWorks and a suite of trained classifiers for taxonomic assignment are also available from GitHub (Table 1). Depending on the DNA metabarcode marker(s) the user will be processing, these can be individually downloaded from GitHub and instructions are provided in the online documentation.

**Table 1:** RDP-trained reference sets that can be used with MetaWorks.

Marker	Target taxa	Classifier availability	Number of included sequences	Number of included taxa at all ranks (species)	Source data
COI	Eukaryotes	https://github.com/terrimporter/CO1Classifier	1,221,528	154,351 (114,687)	BOLD [34], INSDC [35]
rbcL	Diatoms	https://github.com/terrimporter/rbcLdiatomClassifier	3,504	1,432 (1,023)	Diat.barcode [36]
rbcL	Land plants	https://github.com/terrimporter/rbcL_landPlant_Classifier	148,258	61,398 (50,778)	INSDC [35]
rbcL	Eukaryotes	https://github.com/terrimporter/rbcLClassifier	164,454	65,742 (53,344)	INSDC [35]
12S	Fish	https://github.com/terrimporter/12SfishClassifier	2,853	4,751 (2,833)	MitoFish [37]
12S	Vertebrates	https://github.com/terrimporter/12SvertebrateClassifier==	10,654	15,007 (9,564)	INSDC [35] and MitoFish [37]
SSU (18S)	Diatoms	https://github.com/terrimporter/SSUdiatomClassifier	2,962	1,198 (828)	Diat.barcode [36]
SSU (16S)	Vertebrates	https://github.com/terrimporter/16SvertebrateClassifier	72,195	21,282 (15,155)	INSDC [35]
SSU (18S)	Eukaryotes	https://github.com/terrimporter/18SClassifier	42,301	7,504 (5,440 genera)	SILVA [38]
SSU (16S)	Prokaryotes	Built-in to the RDP classifier*	13,212	3,247 (2,506 genera)	RDP [5]
ITS	Fungi (Warcup)	Built-in to the RDP classifier	17,878	10,621 (8,551)	Deshpande et al., 2016 [39]
ITS	Fungi (UNITE 2014)	Built-in to the RDP classifier	145,019	23,222 (20,337)	Abarenkov et al., 2010 [40]
ITS	Fungi (UNITE 2021)	https://github.com/terrimporter/UNITE_ITSClassifier	1,393,203	376,167 (352,588)	UNITE [40]
ITS	Plants	https://github.com/terrimporter/PLANiTS_ITSClassifier	104,387	72,632 (61,693)	PLANiTS [41] and UNITE [40]
LSU	Fungi	Built-in to the RDP classifier	11,442	2,633 (1,895)	Liu et al., 2012 [42]

### Workflow

**Fig 1:**
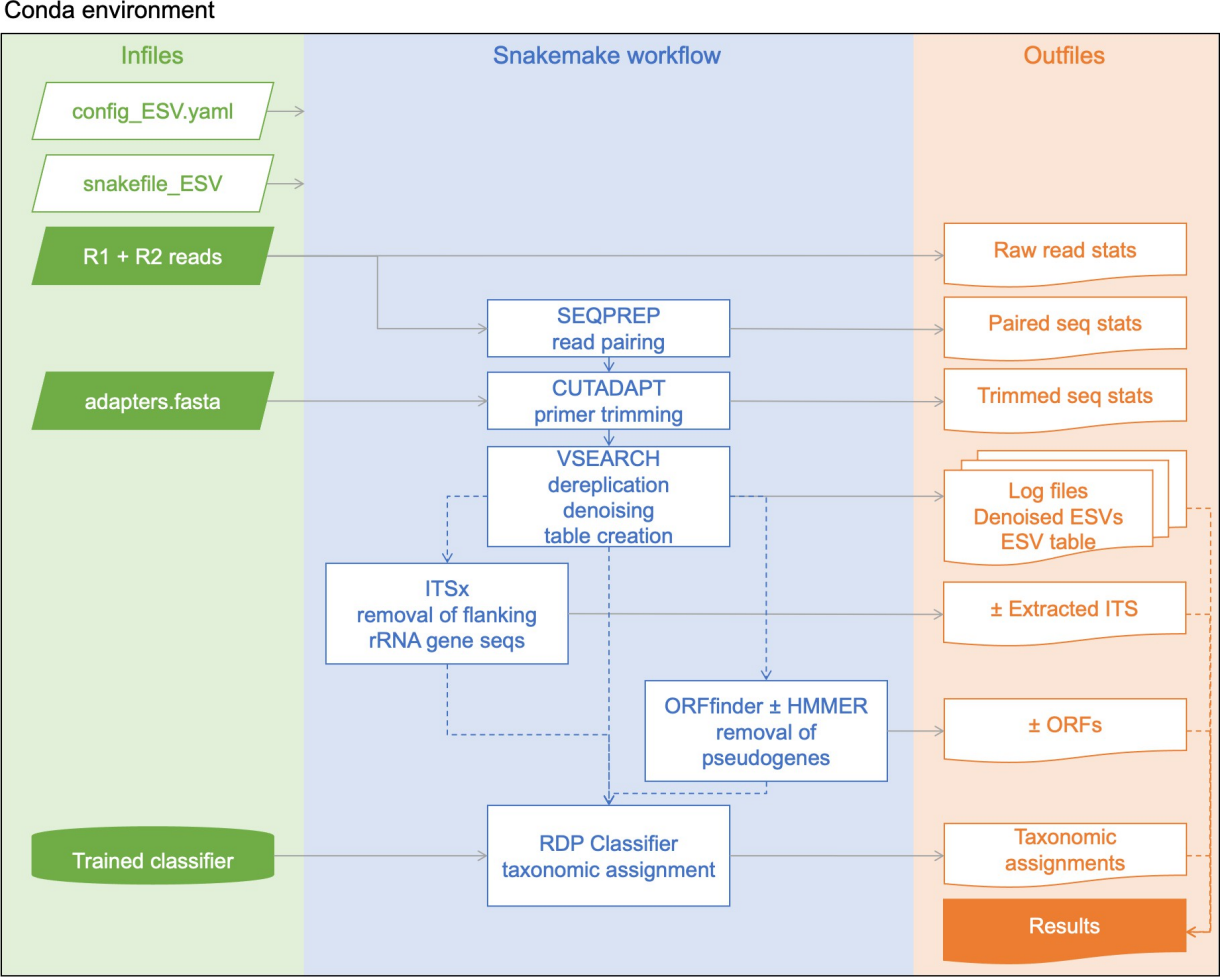
MetaWorks workflow to produce taxonomically assigned exact sequence variants. To aid reproducibility, a Conda environment is provided. Although multiple Snakemake workflows are provided in MetaWorks, here we show the main workflow that generates taxonomically assigned ESVs. Input files are shown in the first panel (green), the ESV workflow is shown in the centre panel (blue), and outfiles are shown in the last panel (orange). The input files in white boxes are required by snakemake to run the appropriate workflow. The input files in green need to be supplied by the user. Note that only custom-trained classifiers such as for COI need to be supplied by the user whereas classifiers built-in to the RDP classifier are used automatically to process prokaryote 16S assignments, for example. The denoising step shown here includes the removal of rare clusters, sequences with putative errors, as well as chimeric sequences. The results are provided in a comma-separated value (CSV) file and shows each ESV per sample with read counts and taxonomic assignments. Abbreviations: Demultiplexed Illumina paired-end reads (R1 + R2), internal transcribed spacer (ITS) region, open reading frame sequences (ORFs).

The pipeline begins with demultiplexed Illumina paired-end reads as this is the format most often provided by sequencing centres to their clients. Several workflows are available as Snakemake pipelines such as taxonomic assignment of ESVs (Fig 1), clustering of ESVs into OTUs, or for processing single reads. For each of these workflows described below, parameter settings for each bioinformatic step can be customized in the config.yaml file. The user also needs to provide a file of primer sequences so we provide a template for the adapters.fasta file as well as a small set of raw Illumina sequences for the COI amplicon that can be used to test the installation. The online documentation provides a tutorial example using the provided COI test data. The tutorial also walks users through the steps necessary to set up their environment to run the pipeline for the first time, assuming the user has never worked with Conda or Snakemake before.

### Exact sequence variants

The ESV workflow will run the pipeline shown in Fig 1. In the config_ESV.yaml file, users indicate the path to the directory that contains the demultiplexed Illumina paired-end reads, specify the unique part of filenames to distinguish between samples and reads, and specify the name of the directory that will contain the outfiles. Default settings for each program are provided in the config_ESV.yaml file but these can be customized by the user. SEQPREP was initially chosen for pairing the forward and reverse reads, because the program comes with the option to output the alignments for visual inspection, an option that most read-pairing programs do not have [43]. For SEQPREP read-pairing, users can specify a Phred score quality cutoff, the minimum overlap between the forward and reverse reads, the maximum fraction of mismatches allowed in the overlap region, and the minimum fraction of matching bases in the overlap region. CUTADAPT was chosen for primer-trimming because it is fast and already widely used in the metabarcoding community for this purpose, so most users will likely already be familiar with how this program works [44]. For CUTADAPT, users need to provide a FASTA-formatted primer sequence file (adapters.fasta), they can also specify the minimum sequence length to retain after primer-trimming, a Phred quality score cutoff, the maximum error rate, minimum adapter overlap, and maximum number of ambiguous bases allowed. VSEARCH was chosen to dereplicate reads (retain unique reads) and remove artefactual sequences using the UNOISE3 and UCHIME3 algorithms [45,46]. We chose the open-source VSEARCH program over alternatives because the program can utilize all the available memory on a system, facilitating the analysis of large datasets on high performance computer systems. We prefer the UNOISE3 method for denoising because it performs up to 1,200 faster and uses less memory than other denoising programs [47]. To map read counts to the newly generated denoised-chimera ESVs to create an ESV x sample table, we use the ‘search_exact’ method because it is faster and optimized to find exact matches compared with the ‘usearch_global’ command with the ‘id 1.0’ parameter, but this is just an intermediate step and further filtering of this table is performed by MetaWorks.

If the internal transcribed spacer (ITS) region is analyzed, then the pipeline uses the ITSx program to trim away the flanking conserved rRNA gene sequences so that taxonomic assignment is based solely on the variable spacer region sequences (ITS1 or ITS2) [19]. This step has been shown to improve sensitivity of clustering and taxonomic assignments [19].

If a protein coding marker is being processed, the user can select a pseudogene-removal method in the config_ESV.yaml file. We have previously described two methods for removing putative pseudogenes from DNA barcode and metabarcoding datasets [21]. The NCBI ORFfinder program is used to translate reads into all possible open reading frames (ORFs). The first pseudogene removal method retains the longest ORF for each read, calculates a distribution of ORF lengths, and removes reads with outlier lengths as putative pseudogenes. The second pseudogene removal method can be used if a hidden Markov model is available and is provided for processing COI arthropods. The longest ORFs are compared to the profile using HMMER available from http://hmmer.org. MetaWorks calculates a distribution of bit scores and removes reads with short outlier bit scores as putative ORFs. Removing noise caused by the sequencing of pseudogenes in metabarcode datasets can help users avoid over-estimating richness in subsequent analyses, yet this step is not included in the most popular metabarcode pipelines as they were developed to support the analysis of rRNA genes where this is not a problem.

One of the features of MetaWorks, is the use of a single taxonomic assignment method for any metabarcode marker that provides a measure of confidence for taxonomic assignments. We chose the RDP Classifier for this task as this method has a long-history of use in the microbial ecology literature, additionally the classifier can be customized and validated for any metabarcode marker [5]. The RDP classifier calculates k-mer frequencies and uses a naive Bayes method to taxonomically assign unknown query sequences. Bootstrapping is used to provide a measure of statistical support, or repeatability, for each assignment at each rank. We have previously described how this method works compared to the top BLAST hit method [28]. In that comparison, we showed how the RDP classifier is faster than the top BLAST hit method and helps to reduce the rate of false-positive assignments. In studies where erroneously identifying a metabarcode sequence as a potential invasive species or pathogen could lead to alarm, reducing the false-positive assignment rate is critical. We provide a suite of trained classifiers, ready for use with MetaWorks (Table 1). Additionally, we provide the training files so that users can check that key target taxa are present in the reference database, and users are free to use the FASTA-formatted sequence files to create custom BLAST databases for similarity-based searches for data exploration or to build reference sets for subsequent phylogenetic analysis. The final file is a comma-separated value file (results.csv) where the taxonomic assignment for each sequence variant is provided for each sample along with read counts. If a rRNA marker was processed, then the ESV sequence is provided in this file; and if a protein coding region was processed using a pseudogene-removal step, then the longest ORF is provided.

### Operational taxonomic units

This pipeline supports the analysis of ESVs for the additional genetic and taxonomic resolution provided by this level of analysis [24]. Though this method of analysis was initially used to process 16S rRNA genes, studies using ITS and COI have also shown that the analysis of ESVs improves the detection of genetic diversity and richness, when assessing beta diversity, both ESVs and OTUs tend to recover similar gradients in multivariate analyses [25,26]. Although it has been shown that for many clustering methods sequence order matters and OTU composition can change from one analysis to the next making reproducibility an issue, there are several reasons why a user would still want to analyze OTUs. For example, it may be more advantageous to work with OTUs instead of ESVs for network analysis to detect more co-occurrences, for legacy reasons to compare results to previous studies that used OTUs, or to approximate ‘species’ units [48].

After processing raw reads using the snakefile_ESV workflow described in the previous section, users can use the snakefile_OTU workflow to cluster ESVs into OTUs. This approach combines the benefits of denoising with clustering using a 97% sequence similarity cutoff using the snakefile_OTU workflow [26,49]. This method uses VSEARCH ‘cluster_smallmem’ method to cluster ESVs using a 97% sequence similarity cutoff. Settings can be adjusted in the in the config_OTU.yaml file such as pointing to the directory that contains the ESVs and choosing a classifier for the OTUs.

## Results and discussion

MetaWorks has already been used in several publications for the Canadian STREAM biomonitoring program, the Government of Canada, Genomics Research and Development Initiative, Metagenomics-based ecosystem biomonitoring (Ecobiomics) project, and by Natural Resources Canada [18,50,51]. The benefits of using an automated, scalable, versioned pipeline for biomonitoring are many-fold, from the ability to share reproducible workflows with collaborators to facilitate the re-analysis of data as more samples are collected from year-to-year. We describe three MetaWorks use-cases in more detail below.

Use case 1: As a part of the Canadian STREAM biomonitoring initiative, the MetaWorks pipeline has been used to process macroinvertebrate COI metabarcodes surveyed from stream sites across Canada [50]. One feature of this project is the quick 1–2 month turn-around time from sampling through to the production of watershed biodiversity reports. This is an improvement over reports generated using conventional morphology-based methods that would normally take 6–12 months to produce. The use of a consistent bioinformatics workflow to process metabarcodes has played a key role in the reproducibility, scalability, and throughput to facilitate timely reporting [52]. Generally, samples are processed in batches of 96 per sequencing run then later split into custom reports for stakeholders, processing about 500 samples per year. One feature of these reports are the taxonomic assignments made using the naive Bayesian classifier that provides bootstrap support values. During the data analysis stage, users can use minimum bootstrap support cutoffs to ensure a certain level of expected accuracy (80–99%) and reduce false-positive taxonomic assignments [28]. The cutoffs used are specific to the amplicon, amplicon length, and taxonomic rank of the assignment and assumes the query is represented in the underlying sequence database. This is in contrast with the use of more traditional methods for taxonomic assignment, where taxa are routinely missed during subsampling and taxa detected by primary analysts and auditors may differ by up to 30% [53]. This use-case shows how MetaWorks can be used to create taxon lists for large-scale biodiversity monitoring of streams across Canada.

Use case 2: Also as part of the STREAM project, MetaWorks results were used to analyze ESVs from diatoms (rbcL) and arthropods (COI) sampled within and across sites of varying water quality [54]. Using the MetaWorks pipeline, two different protein-coding markers were bioinformatically processed in two runs. The first run processed the rbcL marker, using a mixture of 5 different primers in a single adapters.fasta file, and pseudogenes were removed from this dataset using the simple ORFfinder method [21]. The second run processed three COI amplicons, each targeting an approximately 200 bp length of the COI barcoding region using 6 different primers in a single adapters.fasta file, and pseudogenes were removed from this dataset using the ORFfinder+HMMER method since a COI arthropod HMM model was available [21]. The study reported a diversity assessment across sites of varying water quality using richness, effective richness, and beta diversity. Additionally, the taxonomic assignments generated from MetaWorks were used to obtain resource-consumer relationships from a global database of biotic interactions (GloBI) so that community stability using trophic and network measures could be assessed across sites with varying water quality [55]. This use-case shows how MetaWorks can handle a variety of protein-coding markers for trophic and network analyses to facilitate ecological assessments of freshwater condition.

Use case 3: As a part of collaborative work with Environment and Climate Change Canada, MetaWorks was used to assess macroinvertebrate and (non-macroinvertebrate) eukaryote taxa in an urban harbour using COI and 18S rRNA [56]. Using the MetaWorks pipeline, COI metabarcodes were identified down to species rank with 99% accuracy and 18S metabarcodes were identified to genus rank with 80% accuracy using a custom-trained classifier based on the SILVA 18S release 138 [38]. In this study, conventional macroinvertebrate sampling for assessing water quality in Toronto Harbour was compared with metabarcoding methods. COI metabarcoding was found to detect more diversity at a finer level of taxonomic resolution compared with conventional approaches and was able to distinguish sites with particularly high levels of sediment contaminants. Additionally, the use of a multi-marker approach allowed microscopic eukaryote diversity to be sampled at the same time from the same samples, producing indicators that responded to gradients in both sediment contaminants and water physical-chemical features. This use-case illustrates how MetaWorks can facilitate the application of multi-marker metabarcoding approaches that target different domains of life.

As demonstrated in the above examples, MetaWorks supports a wide range of analysis scenarios from metabarcoding data. We envision that MetaWorks will aid broader user communities and fill a need in multi-marker metabarcoding studies that target taxa from multiple different domains of life, to provide a unified processing environment, pipeline, and taxonomic assignment approach for each marker from ribosomal RNA genes, spacers, or protein coding genes. QIIME2 is perhaps the most popular and comprehensive platform for such work, but to date, focuses on processing mainly prokaryote and fungal datasets [16]. To our knowledge, MetaWorks is the only bioinformatic pipeline that can handle rRNA genes but that also integrates special processing steps to handle ITS spacers as well as filter out obvious pseudogenes in protein coding markers such as COI.

There has been a lot of activity with respect to building new bioinformatic tools to handle COI metabarcodes. Recent work, such as the BOLDigger program, makes the BOLD identification engine more suitable for identifying large batches of COI metabaracodes and has both GUI and command-line interfaces for efficient sample processing [57]. A new program, called NUMTdumper, has been developed as a stand-alone program meant to be incorporated into bioinformatic pipelines [20]. NUMTdumper provides a method to screen for NuMTs based on read counts while acknowledging the trade-offs between removing all possible NuMTs while erroneously removing genuine reads. An R package called ‘coil’ has also recently been developed that will place COI barcode and metabarcode sequences in frame using profile HMM analysis [58]. MetaWorks aims to extend the COI metabarcode toolkit that provides a harmonized environment where data from other organismal markers in multi-marker, multi-trophic studies can also be analyzed.

## Conclusion

MetaWorks is provided as free and open software that is versioned, can be deployed in a Conda environment, and is supported by a suite of classifiers for popular metabarcoding markers. The software comes with a small set of raw data and a step-by-step tutorial to help users gain experience quickly. There is extensive online documentation available including detailed explanations of the pipeline, available workflows, and a tutorial for new users who have never used Conda or Snakemake before. MetaWorks generates a CSV file that lists all sequence clusters, for each sample, with associated read counts, taxonomic assignments, and bootstrap support values. Numerous statistics and log files are also provided so that users can track the number of reads that pass each major bioinformatic step. Given the current use of MetaWorks by large-scale national initiatives such as STREAM and Ecobiomics, we foresee additional developments and enhancements. Future planned improvements include the development of additional HMM models for pseudogene filtering, updated and additional classifiers for taxonomic assignment, and support for processing larger jobs both on HPCs and in a cloud environment. We welcome suggestions and potential collaborative work to further advance this pipeline for the scientific community.
